# Occupational stress and social support among nurses

**DOI:** 10.3389/fpubh.2025.1621312

**Published:** 2025-06-18

**Authors:** Beata Dziedzic, Karolina Łodziana, Miłosz Marcysiak, Tomasz Kryczka

**Affiliations:** ^1^Department of Development of Nursing, Social and Medical Sciences, Faculty of Health Sciences, Medical University of Warsaw, Warsaw, Poland; ^2^Graduate, Medical University of Warsaw, Warsaw, Poland; ^3^Department of Social Work, Faculty of Health and Social Sciences, National Academy of Applied Sciences, Ignacy Mościcki in Ciechanów, Ciechanów, Poland

**Keywords:** stress, social support, nurse, occupational stress, work environment

## Abstract

**Introduction:**

Nursing is a profession that involves a significant emotional, physical, and intellectual load. Nurses frequently encounter various stressors, not only due to the nature of their responsibilities but also resulting from organizational factors and interpersonal relationships in the workplace. In this context, social support plays a crucial role in helping individuals cope with stress. It may serve as a protective buffer, promoting positive emotional outcomes and reducing the level of perceived stress. The aim of this study was to analyze stress levels and perceived social support among actively working nurses and to assess the relationship between these two variables.

**Methods:**

The survey included 321 nurses employed at medical facilities. Data collection took place between November 2022 and February 2023. The study was based on a Polish-language survey questionnaire developed through Google Forms, which was distributed via nursing association websites to reach the target audience. Stress levels were measured using the Perceived Stress at Work (PSWP) Questionnaire. Support level was assessed using the Multidimensional Scale of Perceived Social Support (MSPSS).

**Results:**

The average stress level on the PSWP scale was 18.45 ± 5.89, while the average general support score on the MSPSS scale was 63.18 ± 14.78, with the highest support reported from family (21.35 ± 5.3). Analyses revealed a statistically significant negative correlation between MSPSS and PSWP scores (*r* = −0.21; *p* = 0.002).

**Conclusion:**

The findings of this study suggest that most nurses experience moderate levels of perceived stress alongside relatively high levels of social support. Notably, both stress and social support were significantly associated with nurses’ age and workplace setting, indicating that demographic and organizational factors may influence their psychological well-being. These results underscore the need for targeted interventions to reduce stress and strengthen support systems, particularly for younger nurses and those working in high-intensity clinical settings. Further research is warranted to examine causal relationships and to inform the development of tailored support programs within healthcare institutions.

## 1 Introduction

Stress is a significant phenomenon that impacts biology, psychology, physiology, and increasingly, the work environment. However, not all stress responses negatively affect human health. It is currently defined as a state of homeostasis that includes sustress (insufficient stress), distress (harmful stress), and eustress (beneficial stress). Both sustress and distress can have detrimental effects on health, potentially leading to dysfunctional states, while an optimal level of eustress can promote health by restoring homeostasis (1). Occupational stress refers to situations where an individual is overloaded at work, leading to changes in both their mental and physical states, which in turn can affect work performance (2). The above considerations formed the basis for exploring predictors and factors related to occupational stress in nurses (3). Occupational stressors and the factors associated with them can vary depending on the workplace (4) and may change over time due to advancements in healthcare, shifts in interpersonal relationships, and evolving health policies and regulations (5).

In the nursing work environment, there are various stress factors associated with work organization or arising from social interactions (6), as well as individual-level factors, including those related to professional competencies (3, 7). Nursing is widely recognized as one of the most demanding professions in the healthcare sector, characterized by significant emotional, physical, and intellectual burdens. Daily responsibilities, such as patient care, the fast pace of work, the pressure of being responsible for patients’ health and lives, the need to make quick decisions, respond to unforeseen challenges - can all contribute to elevated levels of occupational stress (2, 8–10). Undoubtedly, the daily exposure to the suffering, illness, and death of patients (11) places both physical and mental strain on healthcare workers. This ongoing strain can negatively impact nurses’ work efficiency, thereby potentially compromising patient safety and care quality (12). Occupational stress among nurses stems from excessive workload, such as on-call duties, shift work, or weekend shifts (13), but predisposing factors include insufficient financial compensation (14), unequal treatment in the workplace, lack of recognition for qualifications and opportunities for promotion, job insecurity, and inadequate support from management (11, 15). Occupational stress is also influenced by individual factors such as age, education, and professional experience (3, 7). Negative consequences of occupational stress and reduced job satisfaction can include increased absenteeism (16) and high staff turnover (5). These phenomena may lead to additional costs for healthcare organizations (17) and compromise the quality of care provided to patients (14, 18). Therefore, understanding how stressors impact healthcare professionals is critical, as it enables the implementation of measures to mitigate the stress experienced by nurses and ensure the delivery of high-quality patient care (19).

As a counterbalance to occupational stress, social support is defined as the availability of individuals, groups, or institutions that provide emotional, informational, or instrumental assistance to help individuals cope with difficulties (20). Kiptulon et al. conducted a systematic review of nursing work environments and identified social support as a crucial protective factor that mitigates the adverse effects of stressful work conditions. This support enhances resilience and promotes psychological well-being among nursing staff, which may subsequently improve job satisfaction and reduce the risk of burnout (21).

By addressing the needs arising from stressful events, social support can act as a protective factor by alleviating the level of stress experienced (10, 22). The primary sources of support include family, friends, and close relationships, which can provide emotional, instrumental, and informational assistance (23). Previous studies indicate that even minimal workplace support can significantly reduce the vulnerability of nurses to the adverse effects of stress (24). Such support is linked to improved psychological well-being, increased job satisfaction, and a reduced risk of burnout (24–27). The social support received by nurses plays a key role in helping them adapt to critical events, mitigating the negative impact of stress, and enhancing their ability to cope, thereby increasing their resilience to challenging working conditions (28). According to Cochen et al., interventions implemented to support nurses led to improved well-being, increased work commitment, reduced stress, and decreased burnout, among other benefits. The authors highlight the importance of providing support not only in managing stress but also in addressing its underlying causes (29). Support can be provided through investments in the professional development of employees, which increases job satisfaction and boosts motivation to perform daily tasks, even within a shift-based work structure (30, 31). Research has shown that organizational support contributes to sustaining professional balance and enhancing work-life harmony (32). Positive professional relationships contribute to greater job satisfaction (33), while a well-functioning and supportive work environment with sufficient nursing staff is associated with lower patient mortality rates (34).

In summary, occupational stress impacts the well-being and work performance of nurses, while social support plays a crucial role in mitigating its effects. However, there remains a significant gap in research addressing contemporary issues related to stress and support systems for nurses in Poland. Consequently, this study aimed to examine stress levels and the perception of social support among practicing nurses in Poland, as well as to evaluate the correlation between stress and the perceived availability of social support.

## 2 Materials and methods

### 2.1 Participants

#### 2.1.1 Study design

This cross-sectional observational study was conducted using an online questionnaire developed via the Google Forms platform. The questionnaire, originally prepared in Polish, was distributed electronically through nursing association websites to reach professionally active nurses. A non-probability convenience sampling method was employed, involving voluntary participation of individuals who responded to the survey. An important consideration was the recruitment process: despite broad dissemination, the survey response rate declined substantially during the final phase of data collection. Consequently, data collection was concluded after 4 months, with the final sample size accepted accordingly.

#### 2.1.2 Sample size determination

The required sample size was calculated based on a 95% confidence level, an expected proportion of 0.5, and a maximum margin of error of 5%, resulting in a target sample size of 384 participants.

#### 2.1.3 Study population

According to data published by Statistics Poland (GUS), the number of nurses directly involved in patient care in Poland in 2023 was 216,086 (35). The study targeted nurses employed in healthcare facilities. Inclusion criteria were: age ≥ 21 years, current employment in a healthcare setting, and provision of informed consent. A total of 321 nurses participated in the study.

#### 2.1.4 Ethical considerations

Participation in the study was voluntary and anonymous. Prior to completing the questionnaire, respondents received information regarding the study’s purpose and procedures and were required to provide informed consent. Participants who did not confirm consent were unable to proceed. Withdrawal from the study was permitted at any time without the need to provide a reason, and all previously submitted responses from withdrawing participants were deleted from the system. The study was conducted in accordance with the Declaration of Helsinki and received approval from the Bioethics Committee of the Medical University of Warsaw (Approval No. AKBE/42/2023).

### 2.2 Measures

#### 2.2.1 Sociodemographic data

The sociodemographic questionnaire collected data on gender, age, place of residence, education, work experience, workplace, number of full-time positions, and earnings.

#### 2.2.2 Perceived stress at work scale

The stress level was assessed using the Perceived Stress at Work Scale (PSWS) Questionnaire, in its Polish adaptation by Chirkowska-Smolak and Grobelny (36) based on the PSS-10 scale developed by Cohen and Janicki-Deverts (37). This instrument is designed to subjectively evaluate the extent to which life events are perceived as stressful. The questionnaire items focus on the respondent’s assessment of their beliefs about unpredictability, lack of control, and feelings of being overwhelmed by events. The scale consists of 10 questions, each answered on a 5-point Likert scale, with scores ranging from 0 to 4 points for each response (0 = never, 4 = very often). The total score ranges from 0 to 40 points, with higher scores indicating higher levels of stress. The scores are categorized as follows: low stress (0–13 points), medium stress (14–19 points), and high stress (20–40 points).

In the study Cronbach’s alpha for the PSWS scale was 0.88, indicating good internal consistency. Furthermore, all items exhibited satisfactory correlation with the total scale score.

#### 2.2.3 Multidimensional scale of perceived social support

The level of support was assessed using the Multidimensional Scale of Perceived Social Support (MSPSS), in its Polish adaptation by Buszman and Basista (38) based on the original scale developed by Zimet et al. (39). The questionnaire includes questions designed to examine the functioning of support networks, such as family (4 questions), the respondent’s loved ones (4 questions), and the respondent’s friends (4 questions). The respondents select one answer from a 7-point Likert scale, with scores ranging from 1 to 7 for each response (1 = strongly disagree, 7 = strongly agree). Scores range from 12 to 84 points, with higher values reflecting greater levels of support. The scores are categorized as follows: low support (12–28 points), moderate support (29–57 points), and high support (58–84 points).

In the study, Cronbach’s alpha for the entire MSPSS scale was 0.89, with the following values for the individual subscales: family support 0.90, support from close friends 0.85, and support from friends 0.86.

### 2.3 Data analysis

The data were initially imported into a Microsoft Excel spreadsheet and subsequently transferred to IBM SPSS Statistics for analysis. In the first stage of the analyses, descriptive statistics were calculated, including the mean, median, standard deviation, and minimum and maximum values. For categorical variables, frequency distributions were presented. Due to the violation of assumptions regarding normality of distribution and/or homogeneity of variances, non-parametric tests were applied. Pearson’s correlation coefficient was used to assess relationships between quantitative variables, and in case of assumption violations, Spearman’s rho coefficient was employed. For group comparisons, the Mann–Whitney U test was used for two-group comparisons, and the Kruskal-Wallis test was applied for comparisons involving more than two groups. All analyses were performed with a significance level of α = 0.05. Results were considered statistically significant when the *p*-value was below 0.05.

## 3 Results

The study involved a total of 321 participants, resulting in a response rate of 83.6% of the targeted sample population. Women made up 90.03% of the study group. The largest age group was 21–30 years old, representing 33.04% of respondents. Most participants (50.09%) came from cities with over 500,000 inhabitants, and 44.04% held a bachelor’s degree in nursing. Among the respondents, 32.05% had less than 5 years of professional experience, while 28.04% had over 30 years of experience in the field. The majority of study participants (84.07%) worked in public hospitals. Additionally, 81.03% of respondents were employed in a single full-time position, with the majority reporting earnings between PLN 5,000 and 7,000 (Table 1). Meanwhile, the average gross salary of nurses, ranging from PLN 7,500 to 10,500, is slightly higher than the national average wage (40).

**Table 1:** PSWS and MSPSS scores with regard to sociodemographic variables (*N* = 321).

Parameter	*N*/%		PSWS			MSPSS		
	*N*	%	Mean	SD	*p*	Mean	SD	
Sex								
Female	290	90.03	18.51	5.62	U = 5543.0 *p* = 0.033	63.52	14.76	U = 5211.5 *p* = 0.210
Male	31	9.07	16.09	5.90		60.77	15.01	
Age								
21–30 years	107	33.04	18.87	6.08	H = 5.970 *p* = 0.201	63.85	15.20	H = 3.242 *p* = 0.519
31–40 years	92	11.03	18.66	4.47		64.14	13.45	
41–50 years	69	21.03	18.53	6.08		63.92	14.63	
51–60 years	36	28.08	17.62	5.58		61.78	14.71	
>61 years	17	11.03	16.17	3.53		61.06	15.67	
Place of residence								
Rural	62	19.04	18.02	5.89	H = 1.323 *p* = 0.723	62.34	13.89	H = 1.350 *p* = 0.718
Urban up to 100,000	56	17.02	17.71	6.58		64.02	14.50	
Urban between 101,000 and 500,000	40	12.05	19.15	6.06		63.65	15.01	
Urban > 500,000	163	50.09	18.35	5.17		63.18	14.95	
Education								
Medical high school/medical college	89	27.08	17.95	5.32	H = 0.631 *p* = 0.729	63.10	14.53	H = 0.121 *p* = 0.941
Bachelor of nursing	143	44.04	18.43	5.68		62.87	14.98	
Master of nursing	89	27.08	18.34	6.05		63.90	14.76	
Length of service in the profession								
<5 years	105	32.05	18.61	5.89	H = 2.009 *p* = 0.734	63.95	14.87	H = 0.891 *p* = 0.926
5–10 years	33	10.03	19.27	4.80		61.80	14.11	
11–20 years	71	22.02	18.07	6.21		62.95	15.07	
21–30 years	21	6.06	17.71	5.84		61.52	15.24	
>31 years	91	28.04	17.81	5.29		63.10	14.54	
Workplace								
Public hospital	272	84.07	18.57	5.67	H = 5.358 *p* = 0.252	63.45	14.60	H = 0.980 *p* = 0.913
Private hospital	24	7.05	16.16	5.31		62.20	15.02	
Public clinic	13	4.01	17.23	6.79		62.35	13.85	
Private clinic	8	2.05	16.00	5.21		60.50	14.25	
Nursing and treatment facility	4	1.03	19.00	2.82		64.00	16.10	
Number of full-time positions								
One full-time position	260	81.03	18.25	5.67	H = 0.415 *p* = 0.937	63.35	14.88	H = 0.671 *p* = 0.881
One and a half full-time positions	34	10.03	18.88	5.52		62.90	15.20	
Two full-time positions	26	8.01	17.73	6.23		62.80	14.60	
More positions	1	0.03	17.00	X		60.50	16.30	
Gross earnings								
Up to 5,000 PLN	66	20.03	17.77	5.92	H = 2.451 *p* = 0.484	62.70	14.90	H = 0.554 *p* = 0.907
Above 5,000 to 7,000 PLN	175	54.07	18.48	5.63		63.40	14.50	
Above 7,000 to 10,000 PLN	66	20.06	18.18	6.11		63.10	14.80	
Above 10,000 PLN	14	4.04	16.75	4.99		62.20	15.10	

N, number of observations; %, percent; Mean, average; SD, standard deviation; U, Mann–Whitney U test, H, Kruskal-Wallis H test; p, statistical significance (*p* < 0.05).

A medium level of stress was reported by 56.07% of respondents, while 43.93% indicated receiving a medium level of social support, and 46.72% reported obtaining a high level of support (Table 2).

**Table 2:** PSWS and MSPSS scores.

Scale	Parameter	*N*	%
**Level of stress (PSWS)**	Low	95	29.60
	Medium	190	56.07
	High	46	14.33
**Level of social support (MSPSS)**	Low	30	9.35
	Medium	141	43.93
	High	150	46.72

*N* – Number; % - Percentage.

On the PSWS scale, respondents scored an average of 18.45 ± 5.89 points. On the MSPSS scale, the overall perceived support averaged 63.18 ± 14.78 points, with the highest support reported from family at 21.35 ± 5.3 points (Table 3).

**Table 3:** Descriptive statistics for PSWS i MSPSS.

Scale	Parameter	Min.	Max.	*M*	SD	Med.
**PSWS**	Stress	5	35	18.45	5.89	18
**MSPSS**	General support	12	84	63.18	14.78	66
	Support from a significant other	4	28	20.69	5.76	22
	Support from family	4	28	21.35	5.3	23
	Support from friends	4	28	21.13	5.48	23

Min. - Minimum; Max. - Maximum; *M* – Mean; SD - Standard Deviation; Med. – Median.

Analyses revealed statistically significant correlations between MSPSS and PSWS scale scores. Higher perceived support was associated with lower stress levels. The strongest effect, albeit of moderate magnitude, was observed between the MSPSS total score and the PSWS total score (*r* = −0.21; *p* = 0.0002) (Table 4).

**Table 4:** Correlations between sense of stress and social support.

MSPSS	PSWS	
General support	r Pearsona	*r* = −0.21
	Significance	*p* < 0.05 (0.002)
Support from a significant other	r Pearsona	*r* = −0.20
	Significance	*p* < 0.05 (0.005)
Support from family	r Pearsona	*r* = −0.19
	Significance	*p* < 0.05 (0.005)
Support from friends	r Pearsona	*r* = −0.16
	Significance	*p* < 0.05 (0.003)

The correlation analysis revealed that both age and workplace were significantly associated with the level of perceived social support. A weak negative correlation was found between age and social support (rho = −0.149; *p* = 0.008), indicating that older participants reported lower levels of support. Furthermore, workplace showed significant associations with perceived stress (rho = −0.123; *p* = 0.028) and social support (rho = −0.117; *p* = 0.036), suggesting that different work environments may influence the levels of stress and perceived social support among nurses (Table 5).

**Table 5:** Correlation between the level of perceived stress and social support and sociodemographic variables.

Parameter	PSWS		MSPSS	
	rho	*p*	rho	*p*
Age	−0.0094	0.093	−0.149	0.008
Place of residence	0.006	0.908	0.028	0.621
Education	0.044	0.428	0.111	0.047
Length of service in the profession	−0.034	0.548	0.004	0.948
Workplace	−0.123	0.028	−0.117	0.036
Number of full-time positions	−0.013	0.817	−0.015	0.796
Gross earnings	−0.009	0.874	0.016	0.784

## 4 Discussion

Occupational stress among nurses is a crucial area of research, both for maintaining stable mental health (41), and for ensuring occupational health and safety, as well as delivering high-quality, effective patient care (42). Certain factors, such as advancements in healthcare, regulatory and political changes, and environmental shifts (e.g., pandemics), can contribute to the emergence of occupational stressors among nurses. Therefore, it is crucial to consistently monitor stress levels within this professional group to address these challenges effectively (5).

In the present study, the average stress score was 18.45 ± 5.89, indicating a moderate level. Notably, 66.7% of nurses experienced moderate stress, while 14.33% reported high stress. These findings align partially with trends observed in other Polish studies. Tomaszewska and Kłos (43) found that professional work in healthcare was stressful for 56.25% of the nurses surveyed. Similarly, Kowalczuk et al. (44) reported an average stress level of 16.6 ± 6.1, with the majority of nurses experiencing moderate stress (36.2%). In the study by Kupcewicz et al. (45), 44.89% of nurses reported experiencing moderate stress, while 34.09% reported high stress levels. Another Polish study found that 35.4% of nurses experienced moderate stress, while 14.1% reported high stress (46). Interesting insights emerged from a study conducted among Polish nurses who were offered an opportunity to participate in a refresher course on an innovative wound cleansing method and carried out therapeutic interventions for patients with chronic wounds. Moderate stress levels were reported by 50.7% of nurses, while 36.3% experienced high stress levels. However, the results revealed that nurses who had completed wound care courses, additional training, and specializations, and expressed a willingness to participate in further education - reported significantly lower stress levels compared to others (47). A study conducted during the COVID-19 pandemic revealed that 71.95% of Polish nurses working in public hospitals experienced stress (48). Studies conducted in various healthcare settings have consistently identified stress as a prevalent issue within the nursing profession, with levels varying across countries and work environments. In a study conducted in 2021/2022 among nurses employed in the healthcare systems of eight European countries (France, Germany, Italy, Luxembourg, Portugal, Spain, and Switzerland) 59.3% of nurses reported mild stress, 14.6% reported moderate stress, and 26.1% reported high stress (49). In comparison, in some distant countries like Ghana, 77.8% of nurses experienced moderate levels of stress (50), while in the U. S. (New York) the figure was 64% (51). In Saudi Arabia, 87.6% of nurses reported moderate stress levels (42) and in India the proportion reached 50.8% of nurses (52). These results show that occupational stress poses a significant challenge for the healthcare sector, as the stress experienced by nurses impacts job satisfaction, which may, in turn, affect the quality of patient care (42).

The findings of our study indicate that younger nurses may be particularly susceptible to stress, aligns with the findings of Kowalczuk et al. (44) and Kwiecień-Jagiuś et al. (53), who observed that older nurses tend to manage stress more effectively, often utilizing social support as a coping mechanism.

On the other hand, a correlation was observed between perceived stress levels and the amount of social support received, with lower stress levels reported among nurses who received greater social support. Therefore, our findings highlight the need to implement targeted interventions, such as support programs, to effectively reduce stress levels. This solution is also supported by the research of other authors (54–56).

Social support can thus serve as a buffer, alleviating the negative impact of occupational stress. It can come from colleagues and superiors, offering organizational support (57–59), as well as from family and friends (43, 60). In the present study, respondents reported receiving the highest level of support from their family and friends. Our observations align with the findings of Tomaszewska and Kłos (43) and Śniegocka and Śniegocki (60), where the most commonly reported methods for managing stress were seeking support from friends and engaging in conversations and meetings with loved ones. However, it is equally important that the work environment contributes to strengthening the resilience of nursing staff to stress (45). In their systematic review, Kiptulon et al. emphasized that a supportive organizational culture, characterized by peer collaboration and supervisor support, plays a pivotal role in alleviating occupational stress among nurses. Consequently, implementing strategies to strengthen social support networks within the workplace may be essential for improving nurses’ mental health outcomes and enhancing staff retention (21). Importantly, social support can also be understood through various other sources, such as spiritual leaders, religious communities, sports associations, and other community-based groups. These forms of support often play a crucial role in alleviating stress and enhancing psychological well-being. For instance, spiritual support and active participation in religious communities have been associated with improved health outcomes and reduced stress levels across diverse populations (61).

All the cited studies highlight the critical importance of support, both within and outside the workplace, in assisting nurses to effectively manage occupational stress. The more emotional, informational, and social support nurses receive, the more effectively they cope with stress, leading to improved work performance and better mental and physical health. In light of these findings, it is essential to consistently implement support strategies that focus on developing stress management skills and adopting technologies that aid in stress reduction. Based on our professional experience in clinical settings, we believe that regular meetings with supervisors, access to psychological counseling, and stress monitoring programs can be particularly effective. One notable example is the “Psychological Support Program for Medical Staff” implemented in Poland. This initiative was launched by the Ministry of Health in response to the growing needs of healthcare workers, especially in the context of the COVID-19 pandemic. The program aims to provide rapid, free, and easily accessible psychological support for medical personnel, including nurses, physicians, and other healthcare professionals. It offers, among other services, online consultations with psychologists, telephone support lines, and support groups designed to mitigate the effects of occupational stress, prevent burnout, and improve the mental well-being of medical staff (62).

### 4.1 Strengths and limitations of the study

The study has one notable strength: online surveys tend to yield more reliable data, likely because respondents feel less concerned about their privacy (57). However, this study also has several limitations. The use of an online survey as the data collection method may have influenced both the response rate and the total number of submissions. However, online surveys often yield more reliable data, likely because respondents feel less concerned about their privacy (63). Additionally, the study’s cross-sectional design prevents assessment of changes in stress intensity and support levels over time and limits the ability to draw cause-and-effect conclusions. To gain deeper insights into the dynamics of these relationships, longitudinal studies are needed to monitor how stress and support levels evolve.

This may have been affected by the risk of self-selection bias, limited accessibility of the survey for certain demographic groups (e.g., older adults or individuals with low digital literacy), as well as the inability to clarify ambiguous questions and challenges in ensuring respondents’ full attention while completing the questionnaire (64–66). Moreover, the study was conducted exclusively in Poland, which limits the generalizability of the findings to other countries.

In addition, some of the studies used for comparison employed different survey instruments and collected data across various time periods and countries, complicating direct comparison with the present study. Notably, several of these comparative studies were conducted during the pandemic.

### 4.2 Implications for practice

Given that over half of the respondents reported moderate stress levels, and that perceived social support varied by age and workplace, tailored interventions targeting specific professional groups are essential. The slightly higher levels of support reported by younger nurses suggest a need to enhance support measures for older staff, who may be more vulnerable to insufficient social support.

A key step in reducing occupational stress is the implementation of support programs at both the organizational and individual levels. Evidence from the literature indicates that interventions such as stress management training, relaxation techniques, time management, and work-life balance skills can effectively reduce work-related stress and enhance the well-being of healthcare personnel (67, 68).

Organizational support should include, among other measures, regular meetings with supervisors, opportunities to report work-related challenges, and systemic approaches to workload management. These initiatives help foster a work environment that supports mental health and contributes to improved employee retention (69).

Moreover, the introduction of flexible work schedules can help alleviate stress associated with excessive workloads, as indicated by both the present study and existing literature. Flexibility in work organization has been linked to improved work-life balance, higher job satisfaction, and reduced burnout among nurses (70).

## 5 Conclusion

The study found that most nurses experienced moderate levels of stress alongside relatively high levels of perceived social support. Both stress and social support levels were significantly associated with age and workplace setting. Given the observed associations, future interventions should consider demographic and contextual factors to maximize their effectiveness.

Additionally, the level of social support was significantly correlated with the intensity of stress experienced; individuals receiving greater social support reported lower stress levels. To address the challenges associated with high stress among nurses, it is essential to consistently monitor stress levels and offer targeted educational guidance on effective stress management techniques. Employers and professional organizations should take responsibility for providing this support to create an attractive and sustainable work environment.

The results of the study provide important insights into occupational stress and social support among nurses, highlighting the complex relationships between demographic factors and the work environment. The confirmation of the protective role of social support in alleviating stress reinforces previous studies emphasizing the need for targeted organizational interventions tailored to nurses’ specific needs. Moreover, these findings underscore the importance of integrating stress management strategies into nursing practice and healthcare policy, which may contribute to enhancing nurses’ well-being and the quality of patient care.

In summary, these results not only deepen the understanding of the factors influencing stress among nurses but also provide a practical basis for developing comprehensive support programs to help stabilize the workforce and enhance the functioning of the healthcare system.

## Data Availability

The original contributions presented in the study are included in the article/supplementary material, further inquiries can be directed to the corresponding author.
